# Motor Performance and Skill Acquisition in Oral Motor Training With Exergames: A Pilot Study

**DOI:** 10.3389/fnagi.2022.730072

**Published:** 2022-03-02

**Authors:** Abhishek Kumar, Linda Munirji, Sam Nayif, Nabeel Almotairy, Joannis Grigoriadis, Anastasios Grigoriadis, Mats Trulsson

**Affiliations:** ^1^Division of Oral Diagnostics and Rehabilitation, Department of Dental Medicine, Karolinska Institutet, Huddinge, Sweden; ^2^Department of Orthodontics and Pediatric Dentistry, College of Dentistry, Qassim University, Buraidah, Saudi Arabia

**Keywords:** electromyography, masseter muscle, age groups, complex task, short-term training, biting, mastication

## Abstract

**Objective:**

To investigate the effects of oral-motor training with exergames on motor performance and motor skill acquisition in two different age groups.

**Methods:**

Thirty-two healthy participants were recruited in the current pilot study and divided equally into two groups (Gen Z and Baby Boomers) according to their age. A pair of electromyographic (EMG) electrodes were placed on the participants’ masseter muscles. The EMG device communicated *via* Bluetooth with a mobile video game in response to the electromyographic activity of the masseter muscles during clenching. During the experimental session, participants were asked to play a video game in five blocks of 5 min each, with a 3-min break between each time block. The goal of the game was to collect as many coins (game points) as possible and to dodge/avoid upcoming obstacles (game life). Motor performance was assessed by performance scores and the number of game lives. Skill acquisition was measured by task efficiency (ratio of performance scores and number of game lives) across time blocks.

**Results:**

The results of the study showed significantly lower performance scores (*p* < 0.001), a higher number of game lives (*p* < 0.001), and lower task efficiency in the Baby Boomer group compared to the Gen Z group. Specifically, the results showed that there was a significant difference in task efficiency between the first and second, third and fourth, fourth- and fifth-time blocks in the Gen Z group (*p* < 0.002). However, there was only a significant difference between first- and second-time blocks in the Baby Boomer group (*p* = 1.012), suggesting that skill acquisition in the Baby Boomer group did not change significantly over the course of the time blocks.

**Conclusion:**

The study showed higher motor performance and superior motor skill acquisition with novel exergame training in the Gen Z group compared to the Baby Boomer group. The results of the study indicate that there is an improvement in oral motor skills with short-term training, yet the differences in oral motor skills between the two groups are still evident. The Baby Boomer group, unlike the Gen Z group, did not show robust improvement in task efficiency over the course of the series.

## Introduction

Good oral health is an important aspect of general health and social well-being (Dörfer et al., 2017; Tran et al., 2018). Oral functions such as chewing, bolus formation, and swallowing are important processes that enable optimal food oral processing. Chewing and swallowing functions depend on several factors, including the number of occluding pairs of posterior teeth (Ikebe et al., 2006), type of dentition (Grigoriadis et al., 2011), type and duration of prosthesis use, salivary flow, sensorimotor force regulation during jaw functions (Trulsson, 2006; van der Bilt, 2011; Klineberg et al., 2014; Kumar et al., 2018), etc. In addition, chewing and swallowing functions also depend on the strength of the masticatory, lingual, and other orofacial muscles (Takahashi et al., 2013; Hara et al., 2019).

The age-related loss of muscle mass and strength is widely recognized in the literature (Goldspink, 2012; Keller and Engelhardt, 2013; Larsson et al., 2018). A recent study emphasized that decreased muscle strength may be a major determinant of sarcopenia (Sobestiansky et al., 2019). A previous study in the orofacial area showed that the cross-sectional area of the masticatory muscles decreases with age (Daboul et al., 2018). It is also suggested that aging and sarcopenia not only decrease body strength, but also (adversely) affect tongue pressure, bite force, and swallowing muscle strength (Machida et al., 2017; Kobuchi et al., 2020). Since bite force and tongue motility are essential factors for masticatory function, aging can have a colossal impact on mastication (Peyron et al., 2017).

Strength or resistance training, in general, has been shown to improve muscle strength and endurance (Suchomel et al., 2016). Strength training is an established method to improve a person’s overall muscle mass. It has been shown that chewing and swallowing impairments can be rehabilitated through strengthening exercises of the masticatory muscles (Argolo et al., 2013; Kim et al., 2019). Studies have also shown that oral exercises are effective in improving chewing function (Hakuta et al., 2009) and tongue strength (Oh, 2015; Van den Steen et al., 2018), particularly in people with progressive muscular dystrophy (Kawazoe et al., 1982). However, studies have shown that motivation (Biedenweg et al., 2014) and adherence to traditional exercise paradigms are low in older adults (Simek et al., 2012; Lin et al., 2019).

Recently, in the field of digital games, the term “serious games” has gained widespread use and popularity. The idea of serious games is to integrate games, learning simulations, and/or training for serious purposes such as education, exercise, health, and rehabilitation. Exergaming or training in conjunction with digital games involves playing video games using body movements (Benzing and Schmidt, 2018). Playing a computer game can potentially increase the player’s motivation level during training compared to simple repetitive tasks (Kothari et al., 2013). This is because the “fun” aspect of games keeps the subject interested, unlike other methods that are often considered boring (Hasselmann et al., 2015; Furlan et al., 2019). Exergaming, or exercise combined with active video games, has become an emerging trend in fitness, education, and more recently physical rehabilitation (Pirovano et al., 2016).

Studies have suggested that cognitive and motor abilities vary throughout life (Verhaeghen and Cerella, 2002; Risberg Leversen et al., 2012). Motor performance increases from childhood to young adults and gradually decreases from young adults to old age (Rabbitt et al., 2007; Risberg Leversen et al., 2012). The white matter volume in the brain and age are thought to follow an inverted U-shaped relationship (Sowell et al., 2003). A similar inverted U-shaped curve was also shown between motor performance and age. Accordingly, an increase in motor performance was reported in young adults (19–25 years) and a decrease in relatively older age groups (46–65 years) (Sowell et al., 2003). It has therefore been suggested that the difficulties in acquiring new motor skills in older individuals may be due to declining cognitive abilities with age (Boyd and Vidoni Siengsukon, 2008; Raw et al., 2019). However, although motor performance tends to decline with age, the learning capabilities remain intact (Voelcker-Rehage, 2008). Older individuals are capable of achieving considerable performance gains over a period of time (Voelcker-Rehage, 2008). It has been suggested that task-specific training would increase the probability of neural group formation which may increase the strength of the connections between the neurons and consequently increase motor performance (Risberg Leversen et al., 2012). However, to the best of our knowledge, the potential of exergaming for the training of masticatory muscles and the effect of oral exercises on behavioral learning and skill has not yet been investigated. Therefore, the present exploratory pilot study aimed to investigate the effect of novel oral motor training with exergames on motor performance and motor skill acquisition in two different age groups. We hypothesized that the younger age group would show higher motor performance and improved skill acquisition compared to the older age group.

## Materials and Methods

### Study Participants

The study was conducted in accordance with the Declaration of Helsinki II and approved by the Regional Ethics Review Board in Stockholm, Sweden (Dnr 2018/1963-31). Study participants were students, staff, and volunteers recruited through personal invitations and distribution of flyers on and around the university campus. Volunteers were included in the study (inclusion criteria) only if they were in good general health and had no functional or neurological problems related to biting or chewing (self-reported). Participants were excluded from the study if they showed signs of temporomandibular disorders (TMD) or orofacial pain, as determined by the TMD Screening Questionnaire (Gonzalez et al., 2011). An intraoral examination by the investigator revealed that participants had no current or previous prosthodontic or endodontic treatment and no gross malocclusion of the anterior or posterior teeth, including overjet, overbite, or crossbite. Participants were informed about the methodology of the study and informed consent was obtained prior to participation in the study. The participants were also informed of their right to discontinue the study at any time if they wished. Participants were compensated with a cinema ticket as an appreciation for their participation in the study.

### Experimental Protocol

The experimental session lasted approximately one hour. During the experimental session, participants in both groups were asked to participate in a novel oral motor training session using an exergame. Before the training session began, participants were asked specific questions about the number of hours they spent playing video games and chewing gum. After the training session, participants were asked if they had “fun” during the training exercise. Participants were asked to rate fun on a 0–10 Numerical Rating Scale (NRS) where “0” was no fun and “10” was the most fun ever. In addition, before and after the exercise session, participants indicated how tired they were and how much pain they were in on the 0–10 NRS where “0” was no fatigue and pain and “10” was the highest fatigue and pain imaginable.

### Electromyographic Equipment

During the experimental session, a pair of bipolar electromyographic electrodes (Ambu Neuroline 720, Denmark) was placed on the masseter muscle. A ground reference adhesive electrode with a snap-on connector (OT Bioelettronica, Italy) was also placed on the neck of the participants. Participants with facial hair (beard) were asked to trim/shave or were excluded from the experiment if they were unable to do so. The skin over the masseter muscle and neck was cleansed with alcohol to reduce potential impedance. The portable electromyographic device used in the current experiment was built in such a way that it could communicate with a mobile videogame *via* Bluetooth in response to the electromyographic activity of the masseter muscle during clenching. The masseter muscle was palpated in order to locate the borders and the electrodes were placed parallel to the direction of the muscle fibers in accordance with our previous studies (Kumar et al., 2015a,b, 2017; Grigoriadis et al., 2017; Almotairy et al., 2020). The electrodes were secured with adhesive tapes (Leukoplast BSN medical GmbH, Germany).

### The Novel Oral Motor Training Exercise

**FIGURE 1:**
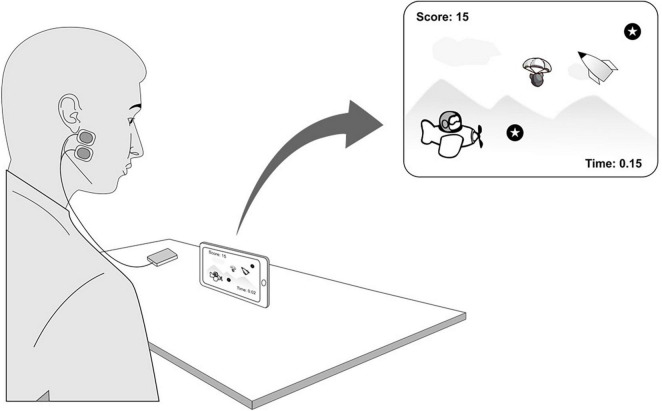
Showing experimental setup with a participant with bipolar electrodes placed on the masseter muscle, the electromyography device and android mobile phone. *Blow-up:* a screenshot of the exergame with the coins (game scores) and obstacles (game life). The electromyographic device could communicate with the mobile videogame *via* Bluetooth in response to the electromyographic activity of the masseter muscle during clenching.

The participants were asked to play an android based video game (OTPlanes, OT Bioelettronica) in five blocks of 5 min each, with a break of three minutes between each time block. The goal of the game was to collect as many coins (game points/score) as possible to achieve the highest possible score while flying a plane (Figure 1). At the same time, the players were supposed to avoid, dodge, and escape the upcoming obstacles (game life) by clenching their jaws. In addition, participants could complete as many trials (game lives) as possible during each time block. Prior to the start of the experiment, participants were shown a small video clip that demonstrated the entire experimental procedure and provided specific instructions on how to play. Note that the participants were not allowed any practice session before the experiment began. Performance was measured as the sum of the game points and the number of game lives during each time block. The higher the total score was, the better the performance, and the greater the number of game lives were, the worse the performance. Further, skill acquisition was measured by task efficiency measured as the ratio of performance scores to game lives across time blocks.

### Statistical Analysis

Data were tested for assumptions of normality using the Shapiro–Wilk test. If the data were not normally distributed, logarithmic transformations were performed. For each participant, the sum of the scores on all trials in each time block was calculated. The total number of game lives during each time block was also counted. The outcome variables (score and number of game lives) were subjected to a two-way repeated-measures analysis of variance (ANOVA) model. The factors in ANOVA were groups (two levels: Gen Z and Baby Boomers) and time blocks (five levels, 1–5). Similarly, task efficiency was calculated by the ratio of game scores and game lives. The data (ratio) was analyzed with two-way ANOVA with repeated measurements. The factors in ANOVA were groups (two levels: Gen Z and Baby Boomers) and time blocks (five levels, 1–5-time blocks). All *post hoc* analyses of the main effects and their interactions were performed with Fisher’s Least Significant Difference test. The data related to the height and weight of the participants were compared with an independent *t*-test. The subject-based reports on fun, fatigue, and pain were evaluated for group differences and differences before and after the training exercises with Mann–Whitney U sign ranked test. A *p*-value of <0.05 was considered statistically significant.

## Results

Thirty-two participants took part in a single experimental session. The participants were evenly divided into Gen Z (*N* = 16, mean age = 21 ± 1.3; age range = 19–23 years, 8 women) and Baby Boomer (*N* = 16, mean age = 62 ± 5.6; age range = 55–77 years, 8 women) groups based on age. All 32 participants were able to complete the entire experimental protocol. Both groups were similar in physical characteristics and there was no significant difference in height, weight, and gender distribution (eight women) between the groups. The participants from both groups rated moderate levels of “fun” in playing the game (Table 1). However, the subject-based reports showed no significant difference in the NRS scores of fun (*U* = 115, *Z* = −0.179, *p* = 0.861), approximate gum chewing (*U* = 88, *Z* = −1.578, *p* = 0.430), and video game playing time (*U* = 105, *Z* = −1.095, *p* = 0.138) between the groups. Further, the results of the ANOVA showed a significant difference in the performance scores between groups and the time blocks which are presented below.

**TABLE 1:** Demographic distribution and Numerical Rating Scale (NRS) scores of participants in both the groups.

	Gen Z	Baby Boomers
Participants (*N*)	16	16
Height	174 ± 8	169 ± 8
Weight	69 ± 11	77 ± 13
Sex	8 women, 8 men	8 women, 8 men
Fun (NRS)	5.8 ± 2.7	5.6 ± 3.9
Gum chewing (h/week)	2.2 ± 1.0	1.7 ± 0.9
Video game (h/week)	1.7 ± 1.0	1.3 ± 0.9

**FIGURE 2:**
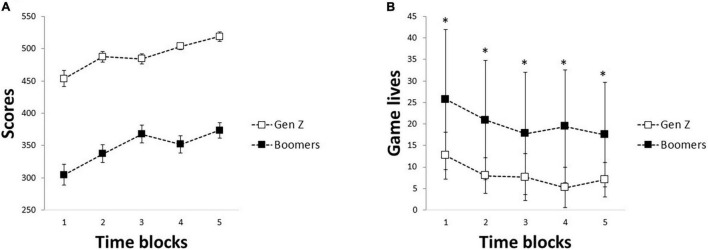
Mean and standard deviation of the performance scores **(A)** and game lives **(B)** across the five-time blocks in the Gen Z (*N* = 16) and Baby Boomer age groups (*N* = 16). Asterisks denote significant interactions between the groups and time blocks (*p* < 0.023).

The results of the two-way repeated-measures ANOVA showed a significant effect of groups [*F*(1) = 21.5, *p* < 0.001, $\eta_{p}^{2}=0.42$, observed power = 0.99] and time blocks [*F*(4) = 7.59, *p* < 0.001, $\eta_{p}^{2}=0.20$, observed power = 0.99] but no significant interaction between the groups and time blocks on the performance scores (Figure 2A). Post-hoc analysis of the time blocks showed significantly higher performance scores during the second to fifth time block as compared to the first-time block and significantly higher performance scores during the fifth time block compared to the second time block.

Similarly, the results of the two-way repeated-measures ANOVA showed a significant effect of groups [*F*(1) = 17.8, *p* < 0.001, $\eta_{p}^{2}=0.37$, observed power = 0.98] and time blocks [*F*(4) = 13.4, *p* < 0.001, $\eta_{p}^{2}=0.31$, observed power = 0.99] on the total number of game lives (Figure 2B). There was also a significant interaction between the groups and time blocks [*F*(4) = 4.8, *p* = 0.001, $\eta_{p}^{2}=0.14$, observed power = 0.94]. *Post hoc* analysis of the interaction showed a significantly higher number of trials (game life) in the Baby Boomers than the Gen Z during all the time blocks (*p* < 0.023).

**FIGURE 3:**
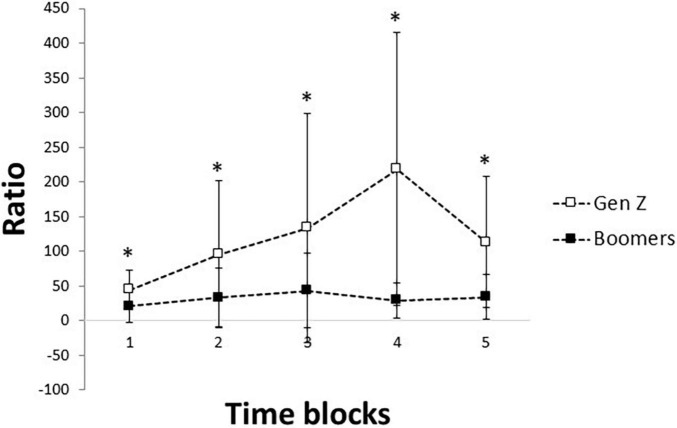
Mean and standard deviation of the task efficiency (ratio of performance scores/game lives) across the five-time blocks in the Gen Z (*N* = 16) and Baby Boomer age groups (*N* = 16). Asterisks denote significant interactions between the groups and time blocks (*p* < 0.05).

The ratio of the performance scores and number of games lives (i.e., task efficiency) was assessed as a measurement of skill. The result of the ANOVA showed a significant effect of groups [*F*(1) = 24.7, *p* < 0.001, $\eta_{p}^{2}=0.45$, observed power = 0.99] and time blocks [*F*(4) = 19.2, *p* < 0.001, $\eta_{p}^{2}=0.38$] and a significant interaction between the groups and time blocks [*F*(4) = 4.6, *p* = 0.002, $\eta_{p}^{2}=0.13$, observed power = 0.94] (Figure 3). *Post hoc* analysis of the interaction showed significantly higher task efficiency in the Gen Z group than the Baby Boomer group during all the time blocks (*p* < 0.001). Also, there was a significant difference in the task efficiency between the first and second, third and fourth, fourth- and fifth-time blocks in the Gen Z group (*p* < 0.002). However, there was only a significant difference between first- and second-time blocks in the Baby Boomer group (*p* = 0.012), indicating that the Baby Boomer group did not show a robust change in task efficiency as the series progressed.

**FIGURE 4:**
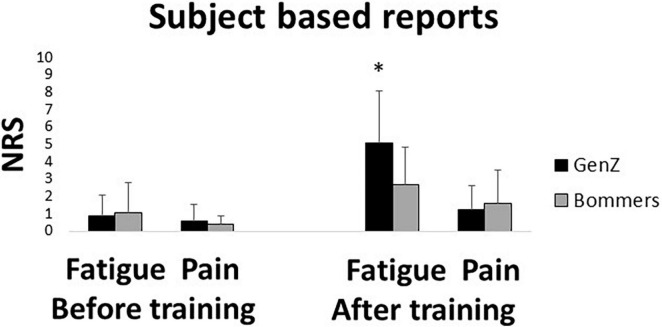
Mean and standard deviation of the subject-based reports on fatigue and pain, before and after the training exercises in the Gen Z (*N* = 16) and Baby Boomer age groups (*N* = 16). Asterisks denote significant differences between the groups (*p* = 0.026).

The subject-based reports showed no significant difference in the fatigue (*U* = 120, *Z* = −0.306, *p* = 0.780) and pain (*U* = 127, *Z* = −1.089, *p* = 0.985) NRS scores between the groups before the training exercises. However, the Gen Z group reported significantly higher fatigue scores (*U* = 69, *Z* = −2.245, *p* = 0.026) than the Baby Boomer group after the training exercises. There was also no significant difference in the pain scores between the groups after the training exercises (*U* = 111, *Z* = 0.723, *p* = 0.740). On comparing the NRS scores before and after the training exercises, both the groups reported significantly higher fatigue (Gen Z: *Z* = −3.075, *p* < 0.001; Baby Boomer: *Z* = −2.322, *p* = 0.020) and pain (Gen Z: *Z* = −2.141, *p* = 0.016; Baby Boomer: *Z* = −2.120, *p* = 0.034) scores after than before the training exercises (Figure 4).

## Discussion

The aim of the current exploratory pilot study was to investigate the effect of a novel oral motor training on motor performance and motor skill acquisition in a younger (Gen Z) and a relatively older (Baby Boomers) adult. The results of the study showed higher motor performance and superior motor skill acquisition in the Gen Z group compared to the Baby Boomer group. Specifically, the results showed higher performance scores, a fewer number of game lives, and greater task efficiency in the Gen Z group compared to the Baby Boomer group. In addition, the Gen Z group showed significant effects of training reflected in improved task efficiency across time blocks, whereas there were no such robust effects of training in the Baby Boomer group.

### Motor Performance

Our results showed a clear difference in oral motor performance between the two groups. It was also found that the participants’ physical characteristics (i.e., height and weight), motivation, fun, and average exposure to similar tasks (i.e., playing a video game) were comparable in the two groups. While there is a possibility that the difference in motor performance could be dependent on other factors, one important distinction was the age of the participants in the two groups. In the current study, the Baby Boomer group was in the age range of 55–77 years and performed worse in oral motor training than the younger Gen Z group (age range = 19–23). Therefore, the difference in motor performance in the current study to a large extent may be attributed to an age-related decline in oral motor skills. Our findings are supported by the results from a recent systematic review that showed changes in bite force with age. Specifically, the study showed a decreasing trend in bite force at age 50 years (Almotairy et al., 2018). Hence, the age-related decrease in sensorimotor regulation of bite force may be responsible for the between-group differences in motor performance in the current study. However, our results are in contradiction with the findings of a previous study indicating similar motor performance in both young and older adults in isometric force control tasks (van Dijk et al., 2007). Motor performance may be influenced by several general factors such as participants’ physical characteristics, age, sex, as well as specific factors such as the novelty and complexity of the motor task, fatigue, motivation, and active participation (Wulf et al., 2010). The influence of factors such as the novelty and complexity of the motor task on motor performance and skill acquisition is discussed below.

### Motor Skill Acquisition

In the current study, we observed robust effects of training in the Gen Z group. These robust effects were reflected in increased task efficiency between the first and second, third and fourth, fourth- and fifth-time blocks. In contrast, the Baby Boomer group showed only a modest effect of training, reflected in a significant difference in task efficiency between the first- and second-time blocks. These results indicate that the Gen Z group showed better task efficiency and thus a higher skill acquisition compared to the Baby Boomer group. Our results are consistent with the findings from a previous study that showed that young adults performed better in tongue training tasks and showed higher skill acquisition than older individuals (Kothari et al., 2014). In contrast, it was also shown that there was no apparent effect of training on jaw function during repetition of a simple biting task (Kumar et al., 2014). However, these differences could be due to factors related to the complexity of the task (see below). The acquisition of motor skills is a learning process in which repetition of a task leads to permanent changes in an individual’s ability to perform a specific task (Araújo et al., 2004). Our results are also consistent with the findings of previous studies suggesting that aging is associated with significant changes in the sensorimotor system, leading to a marked deterioration in motor skill acquisition in older individuals (Voelcker-Rehage, 2008; Raw et al., 2019).

### Novelty and Complexity of the Task

The oral motor task (exergame) in the current study was novel to all participants in both groups. While computer gaming consoles are typically operated and controlled with the hands/digits, they have not been fairly adapted to be controlled with the jaw muscles. Despite the novelty, the results of the study showed distinct differences in motor performance and skill acquisition between the two groups. Furthermore, the oral motor task in the current study was quite complex compared to simple biting tasks. The motor task, which involved controlling the movement of the (video) game specimen by skillfully regulating the biting and clenching forces while collecting game points (performance scores) and avoiding obstacles (game lives) was novel to the participants in both the groups. It is agreed that age-related motor performance is more pronounced in complex tasks compared to simple tasks (Voelcker-Rehage, 2008). Complex motor tasks depend on several physiological mechanisms that include sensorimotor integration, attentional processing, and performance monitoring (Alahmadi et al., 2016). It has been shown that complex tasks are more likely to show age-related differences compared to simple tasks (Voelcker-Rehage, 2008). Because the motor task in the current study was novel and quite complex, our results showed significant differences in motor performance and motor skill acquisition between the two groups.

### Subject-Based Reports

The participants in the Gen Z group reported moderate levels of fatigue and showed higher performance scores than the Baby Boomers’ group. These reports are consistent with previous studies in which participants reported moderate levels of fatigue after tongue training exercises (Svensson et al., 2006; Kothari et al., 2012). Fatigue and pain are strong factors that can influence motor performance (Boudreau et al., 2010). The participants in the Baby Boomer group were less fatigued because they may have been more careful in performing the task. In general, it has been observed that the older individuals tend to perform motor tasks cautiously and sometimes less accurately because of a general decline in sensorimotor regulation. It is suggested that the “cautiousness” may have led not only to a decrease in performance scores, but also to less fatigue compared to the Gen Z group (Enoka and Duchateau, 2016).

### Clinical Relevance and Future Perspectives

The results of the current pilot study show a significant difference in motor performance on the exergame task between the two age groups. The results of the study imply that the exergame exercise has the potential (see results for observed power) to differentiate motor performance between a younger and an older age group. Hence, it is suggested that the exergame task can be used as a tool to examine/study oral motor performance. The results of the current study may be beneficial because skilled motor performance is an important aspect of (oral) rehabilitation interventions. Motor skills play an important role throughout life, and therefore older adults also need to learn or relearn motor skills as part of training for new tasks in everyday life or rehabilitation. Therefore, motor performance and skill acquisition can be important factors influencing healthy living and the implementation of therapeutic approaches to rehabilitation, especially in older individuals (Ehsani et al., 2015). Combining the appeal of gameplay with rehabilitating exercise creates an opportunity for increasing compliance to exercises paradigms. However, the extent to which these findings translate to the clinical setting needs to be explored in future studies. The results also indicated that the Baby Boomer group did not show robust signs of skill acquisition due to short-term training. Nevertheless, it can be anticipated that the Baby Boomer group may increase motor performance and motor skill acquisition if the training is prolonged, perhaps over several days or weeks in accordance with the previous suggestion (Kumar et al., 2014). Hence, future studies may also aim to investigate whether longer/more training sessions or the influence of video game experiences on oral motor performance and skill acquisition. Future studies also need to confirm if the exergame task could develop as an exercise tool to improve oral functions. Conventional oral/jaw exercises at times may not persuade patients to complete the intended training because they do not maintain the individual’s interest. If the expected results from the future studies are achieved substituting exergames for traditional training could increase patient compliance and motivate them to achieve better training results.

## Conclusion

Short-term oral motor training with exergames showed differences in oral motor performance between the two groups. In addition, the results also showed differences in oral motor skill acquisition between the two groups with the younger group showing signs of higher motor performance and higher skill acquisition compared to the older age group. It is therefore suggested that more training efforts may be needed to train individuals in the older age group. Overall, the results of the study enhance our understanding of the oral motor system and interindividual differences in oral motor skills between the two age groups. These findings may contribute to a better understanding of the effects of oral exercise on training-induced neuroplasticity and the acquisition (or re-learning) of new motor skills.

## Data Availability Statement

The raw data supporting the conclusions of this article will be made available by the authors, without undue reservation.

## Ethics Statement

The studies involving human participants were reviewed and approved by the Swedish Ethical Review Authority (Etikprövningsmyndigheten). The patients/participants provided their written informed consent to participate in this study.

## Author Contributions

AK, AG, and MT conceived and designed the study, and finalized the draft. AK, LM, and SN collected the data. AK, NA, and JG analyzed the preliminary data and designed the figures. AK and NA performed the statistics. AK drafted the first draft and edited several versions of the manuscript together with all authors. All authors substantially contributed and approved the final version of the manuscript.

## Conflict of Interest

The authors declare that the research was conducted in the absence of any commercial or financial relationships that could be construed as a potential conflict of interest.

## Publisher’s Note

All claims expressed in this article are solely those of the authors and do not necessarily represent those of their affiliated organizations, or those of the publisher, the editors and the reviewers. Any product that may be evaluated in this article, or claim that may be made by its manufacturer, is not guaranteed or endorsed by the publisher.
